# A pathogenic variant of TULP3 causes renal and hepatic fibrocystic disease

**DOI:** 10.3389/fgene.2022.1021037

**Published:** 2022-10-07

**Authors:** Hossein Jafari Khamirani, Vivek Reddy Palicharla, Seyed Alireza Dastgheib, Mehdi Dianatpour, Mohammad Hadi Imanieh, Seyed Sajjad Tabei, Whitney Besse, Saikat Mukhopadhyay, Karel F. Liem

**Affiliations:** ^1^ Department of Medical Genetics, Shiraz University of Medical Sciences, Shiraz, Iran; ^2^ Department of Cell Biology, University of Texas Southwestern Medical Center, Dallas, TX, United States; ^3^ Stem Cells Technology Research Center, Shiraz University of Medical Sciences, Shiraz, Iran; ^4^ Gastroenterohepatology Research Center, Shiraz University of Medical Sciences, Shiraz, Iran; ^5^ Shiraz Nephro-Urology Research Center, Shiraz University of Medical Sciences, Shiraz, Iran; ^6^ Department of Internal Medicine, Section of Nephrology, Yale School of Medicine, New Haven, CT, United States; ^7^ Vertebrate Developmental Biology Program, Department of Pediatrics, Yale University School of Medicine, New Haven, CT, United States

**Keywords:** cilia, tubby domain, cyst, fibrosis, PKD, liver, ciliopathy, kidney

## Abstract

Patient variants in *Tubby Like Protein-3 (TULP3)* have recently been associated with progressive fibrocystic disease in tissues and organs. TULP3 is a ciliary trafficking protein that links membrane-associated proteins to the intraflagellar transport complex A. In mice, mutations in Tulp3 drive phenotypes consistent with ciliary dysfunction which include renal cystic disease, as part of a ciliopathic spectrum. Here we report two sisters from consanguineous parents with fibrocystic renal and hepatic disease harboring a homozygous missense mutation in *TULP3* (NM_003324.5: c.1144C>T, p.Arg382Trp). The R382W patient mutation resides within the C-terminal Tubby domain, a conserved domain required for TULP3 to associate with phosphoinositides. We show that inner medullary collecting duct-3 cells expressing the TULP3 R382W patient variant have a severely reduced ability to localize the membrane-associated proteins ARL13b, INPP5E, and GPR161 to the cilium, consistent with a loss of TULP3 function. These studies establish Arginine 382 as a critical residue in the Tubby domain, which is essential for TULP3-mediated protein trafficking within the cilium, and expand the phenotypic spectrum known to result from recessive deleterious mutations in *TULP3*.

## Introduction

Primary cilia are small signaling organelles that project from the surface of nearly all cells of the human body. Mutations in genes that affect cilia are at the origin of a group of human genetic diseases referred to as “ciliopathies.” Disease phenotypes caused by mutations in cilia-related genes are characterized by an overlapping spectrum of multisystem disorders which include renal cystic disease, skeletal malformations, neural developmental defects and retinal degeneration, among others (Goetz and Anderson, 2010; Reiter and Leroux, 2017). Many genes are involved with building a functional cilium and the severity and nature of the disease resulting from their mutations depend on the individual gene affected and the nature of the mutation.

Recently, variants in the *Tubby like protein-3 (TULP3)* have been associated with multisystem fibrotic disease in patients (Devane et al., 2022). TULP3 (Tubby like protein-3; MIM: 604730) encodes a member of the Tubby domain family of proteins (Mukhopadhyay and Jackson, 2011) which contain a signature carboxy-terminal Tubby domain that functions to bind selectively to membrane phosphoinositides (Santagata et al., 2001; Mukhopadhyay et al., 2010). In addition, the TULP3 amino terminus contains a short, conserved domain that strongly binds proteins of the Intraflagellar transport complex A (IFT-A) (Mukhopadhyay et al., 2010), an essential protein complex required for the trafficking proteins in and out of the cilium (Liem et al., 2012). In the mouse, deletion or strong loss of function alleles of *Tulp3* are associated with embryonic lethality, neural tube defects, and limb patterning defects (Ikeda et al., 2002; Cameron et al., 2009; Norman et al., 2009; Patterson et al., 2009). Unlike most ciliary mutations, *Tulp3* mutations do not cause major structural defects in cilia (Norman et al., 2009; Hwang et al., 2019; Legue and Liem, 2019; Legue and Liem, 2020). *Tulp3* phenotypes have been shown to cause a gain of function in the Sonic Hedgehog (Shh) pathway in mutant mice, which has been shown to be Gli dependent (Norman et al., 2009; Legue and Liem, 2020). Changes in Shh signaling pathways affect multiple developmental pathways and underlie a subset of the phenotypes of the ciliopathic spectrum.

Here we present two sibling patients from a consanguineous Iranian family presenting with fibrocystic renal and hepatic disease and harboring a homozygous *R382W TULP3* mutation. We have modeled the effect of the mutation on TULP3 function *in vitro* and show that the amino acid substitution acts to disrupt protein trafficking to the primary cilium. Our results show that the mutation causes a loss of function consistent with a disruption of the conserved Tubby domain. These results identify R382 as a critical residue for TULP3 function and expand our knowledge of recessive fibrosis-associated disease mutations in patients.

## Materials and methods

### Patient identification

**FIGURE 1:**
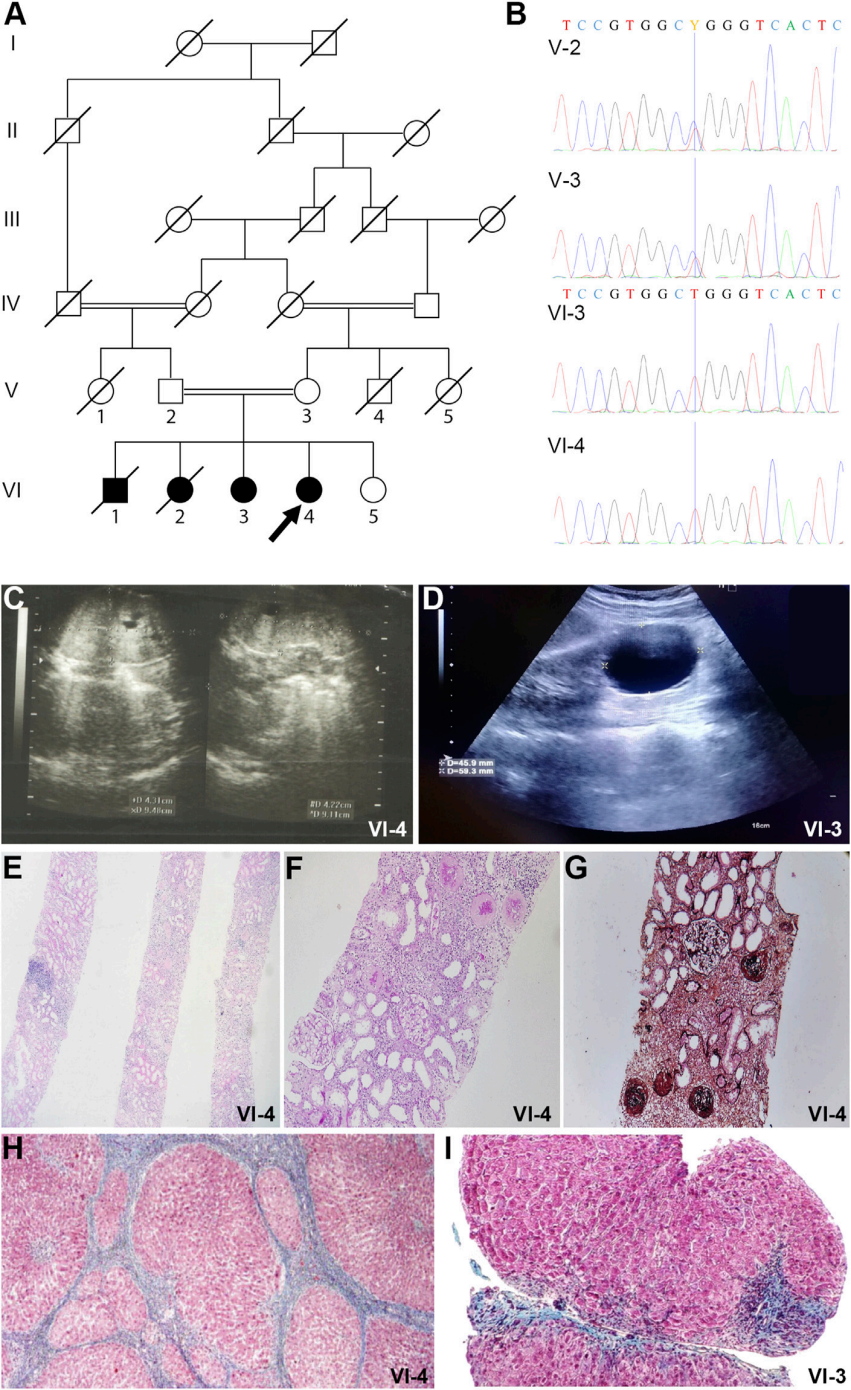
Pedigrees and imaging studies of *TULP3* patients. **(A)**. Pedigree of the family. The pedigree of the family illustrates the recessive inheritance of the pathogenic variant in the family. Double lines in pedigrees indicate consanguinity. Filled and unfilled circles/squares represent affected and unaffected individuals respectively, while circle/squares with diagonal lines indicate deceased individuals. Proband is VI-4 (arrow). **(B)** Electropherogram of the family. The homozygous variant in the proband (individual VI-4) and her sister (individual VI-3). The heterozygous variant in their parents (individual V-3 and V-4). Missense variant c.1144C>T results in alteration of Arginine to Tryptophan in position 382 of the TULP3 protein. **(C)** Abdominopelvic Sonography of patient VI-4. Kidney ultrasonography of the patient shows enlarged kidneys and several small cysts. **(D)** Abdominopelvic Sonography of patient VI-3. There is a simple cortical cyst measuring 45 × 59 mm in the mid pole of the left kidney. **(E)** Kidney core needle biopsy of patient VI-4. Histology shows diffuse global glomerulosclerosis, tubular atrophy and moderate interstitial infiltration of mononuclear inflammatory cells (H&E stain, magnification ×40). **(F)** Kidney core needle biopsy of patient VI-4. PAS stain, magnification ×100. **(G)** Kidney core needle biopsy of patient VI-4. Jones silver stain, magnification ×100. **(H)** Section from liver needle biopsy of patient VI-4. Masson-Trichrome stain shows bridging fibrosis and nodule formation and distorted lobular and vascular architecture with micronodule formation. Individual hepatocytes show ballooning degeneration. **(I)** Section from liver needle biopsy of patient VI-3. Masson-Trichrome stain shows distorted lobular and vascular architecture with micronodule formation. Individual hepatocytes show ballooning degeneration. Marked bridging fibrosis is present with occasional nodules portal spaces.

The proband (VI-4) is a 12-year-old female with a suspected inherited hepatic renal syndrome. The proband is from an Iranian family with four affected children born to first-degree consanguineous parents (Figure 1A). Following clinical and genetic investigation, the family consented to written informed approval for participation in this study and publication of the results.

### Genetic studies

DNA from peripheral blood samples were collected from the subjects for Whole Exome Sequencing (WES) and Sanger Sequencing using QIAamp DNA Blood Mini Kit (Qiagen). WES identified a homozygous variant (NM_003324.5:c.1144C>T, p.Arg382Trp) of *TULP3* in the proband. The missense variant (NM_003324.5:c.1144C>T, p. Arg382Trp) detected by WES in *TULP3* is classified as “ Likely Pathogenic” based on the PM2, PP3, and PS3 criteria of the ACMG/AMP guidelines (Richards et al., 2015). It has a minor allele frequency of 1.6 × 10-5 in gnomAD (Karczewski et al., 2020). The variant was classified as a “disease-causing,” “pathogenic,” or “damaging” based on pathogenicity prediction tools including MetaLR, MetaSVM, MetaRNN, REVEL, BayesDel addAF, BayesDel noAF, CADD, DEOGEN2, EIGEN, FATHMM, LIST-S2, LRT, M-CAP, MutPred, MVP, Mutation Assessor, MutationTaster, PROVEAN, Polyphen, PrimateAI and SIFT.

### PCR amplification and Sanger Sequencing

The primers were designed using Oligo Primer Designer (Rychlik, 2007). Forward primer (5′- CTT​CTG​GTT​TAC​CCT​TTC​TG), reverse primer (5′- ATG​ATT​ATC​TGC​TGA​CTG​TC). The presence of the variant in the tenth exon of the TULP3 gene was confirmed by Sanger sequencing.

### Protein structure

Using AlphaFold Protein Structure Database, the predicted three-dimensional structure of TULP3 protein based on amino acid sequence and the position of the pathogenic variant in this study was modeled. Domains of Tubby-Like Protein 3 in the two-dimensional structure were collected from InterPro. The two-dimensional and three-dimensional structures of TULP3 protein illustrate the position of the variant identified in this study.

### Expression constructs

pENTR221-INPP5E construct was from Life technologies (IOH40212). Retroviral constructs of HA-INPP5E were generated by cloning into pQXIN-HA vector. pG-LAP1 (pCDNA5/FRT/TO-EGFP-TEV-Stag-X) was from Addgene (Torres et al., 2009). N-terminal LAP-tagged retroviral constructs of full length and mutant TULP3 were generated by Gateway cloning into a gateway LAP1 version of pBABE. Retroviral constructs of HA-INPP5E were generated by cloning into pQXIN vector. TULP3 R382W was generated by Q5 site-directed mutagenesis (New England Biolabs).

### Cell culture and generation of *Tulp3* knock out and stable cell lines

CRISPR/Cas9 knockout lines for *Tulp3* were generated in IMCD3 Flp-In (Invitrogen) cells by cloning targeting sequences in exon 3 of mouse *Tulp3* (ACG​TCG​CTG​CGA​GGC​ATC​TG and TGG​CTT​TAA​CCT​TCG​CAG​CC) into pLentiCRISPR backbone. Single clones were isolated using serial dilution method. Clonal lines were tested for knockout by Sanger sequencing and immunoblotting for Tulp3 (Palicharla et al., 2021). Stable lines expressing N-terminal LAP-tagged full length or mutant TULP3 and HA-INPP5E were generated by retroviral infection and antibiotic selection. IMCD3 cells were cultured in DMEM high glucose (Sigma-Aldrich; supplemented with 10% cosmic serum, 0.05 mg/ml penicillin, 0.05 mg/ml streptomycin, and 4.5 mM glutamine).

### Immunofluorescence of cultured cells and microscopy

Cells were cultured on coverslips until confluent and starved for indicated periods. Cells were fixed with 4% PFA. After blocking with 5% normal donkey serum, application of primary antibody was for 1 h, and secondary antibodies for 30 min with Hoechst 33,342 (Invitrogen). Primary antibodies used were Acetylated tubulin (T6793); ARL13B (Caspary et al., 2007), HA-tag (3F10, Roche), and GPR161 (Pal et al., 2016). Secondary antibodies were from the Jackson Immuno. Coverslips were mounted using Fluoromount G (SouthernBiotech). Images were acquired on a microscope (AxioImager.Z1; ZEISS), sCMOS camera (PCO Edge; BioVision Technologies), and Plan Apochromat objectives (×40/1.3 NA oil; and 63×/1.4 NA oil) controlled using Micro-Manager software (UCSF). Between 8 and 20 z sections at 0.5–0.8-µm intervals were acquired. For quantitative analysis of ciliary localization, stacks of images were acquired from three to eight consecutive fields and percentages of protein-positive ciliated cells were counted. Maximal projections from stacks were exported from ImageJ/Fiji (NIH) using a custom-written macro (M. Mettlen, UT Southwestern) using similar parameters for image files from the same experiment. For measuring ciliary pixel intensities, image stacks were acquired with z sections at 0.8-µm intervals. An image interval with maximal intensity was chosen, and cilia were demarcated with a region of interest using the fluorescence signal for acetylated *α*-tubulin. The mean pixel intensities for the corresponding protein were exported from ImageJ/Fiji.

### Statistical analyses

Statistical analyses were performed using ANOVA and Tukey’s post hoc multiple comparisons between all possible pairs using GraphPad Prism.

## Results

The proband in this study is a twelve-year old female, the fourth child from a healthy consanguineous Iranian couple (Figures 1A,B, individual VI-4, arrow). The couple reported that their first two children died at the age of 2 years (VI-1) and at 3 months (VI-2) and they were told that the cause of death was kidney failure, but they knew no further information, and no specimen was available for subsequent genetic investigation. She (VI-4) was born following full-term pregnancy, however given her sibling history, she had an abdominal ultrasound within days after her birth. This revealed enlarged kidneys with many small cysts. Subsequent ultrasounds over the first 4 years of her life showed more prominent size of cystic kidneys and increased corticomedullary echogenicity, with reference to grade 1 hydronephrosis in some studies (Figure 1C). Polyuria was reported. Serum Creatinine was 1.8 mg/dl by age 4. A kidney core needle biopsy at age 5 showed diffuse global glomerulosclerosis and tubular atrophy with tubular dilations (Figures 1E–G). The patient underwent a kidney transplantation at age 5, which continues to function well.

Ultrasonography initially described a normal appearing liver, however concern was raised about the liver at age 4 based on elevated SGOT and SGPT, low ferritin, and abnormal coagulation parameters. Subsequent liver ultrasounds from age 5 to age 11 showed initially a mildly enlarged liver with heterogeneous echogenicity, but subsequently smaller in size with lobulated border suggestive of cirrhosis, and with persistent splenomegaly (19.9 cm). A needle biopsy of the liver showed distorted lobular and vascular architecture with micronodule formation with individual hepatocytes showing ballooning degeneration. Masson-Trichrome showed bridging fibrosis and nodule formation (Figure 1H).

The proband’s older sister was also evaluated for fibrocystic kidney and liver disease (Figure 1A, individual VI-3). Ultrasonography revealed a simple kidney cyst that has grown to 4.5 cm × 5.9 cm (Figure 1D). She has urine volumes in the range of 2 L per day. Her liver function and appearance have been abnormal similar to that of the proband. At age ten years, a liver needle biopsy revealed distorted lobular and vascular architecture with micronodule formation with individual hepatocytes showing ballooning degeneration (Figure 1I).

Whole exome sequencing of the proband’s genomic DNA was performed and a novel homozygous variant in *TULP3* was identified and verified by Sanger sequencing. The identified homozygous missense mutation (NM_003324.5: c.1144C>T, p. Arg382Trp) in *TULP3* was present as a heterozygous variant in both unaffected parents (Figures 1A,B). Both the proband and her affected sibling harbored this homozygous mutation while their unaffected sibling did not carry the mutation. Two additional deceased siblings who had hepatorenal disease were not analyzed in this study (Figure 1A). See Supplementary Material S1 for a homozygosity plot of the proband and Supplementary Material S2 for additional variant information.

**FIGURE 2:**
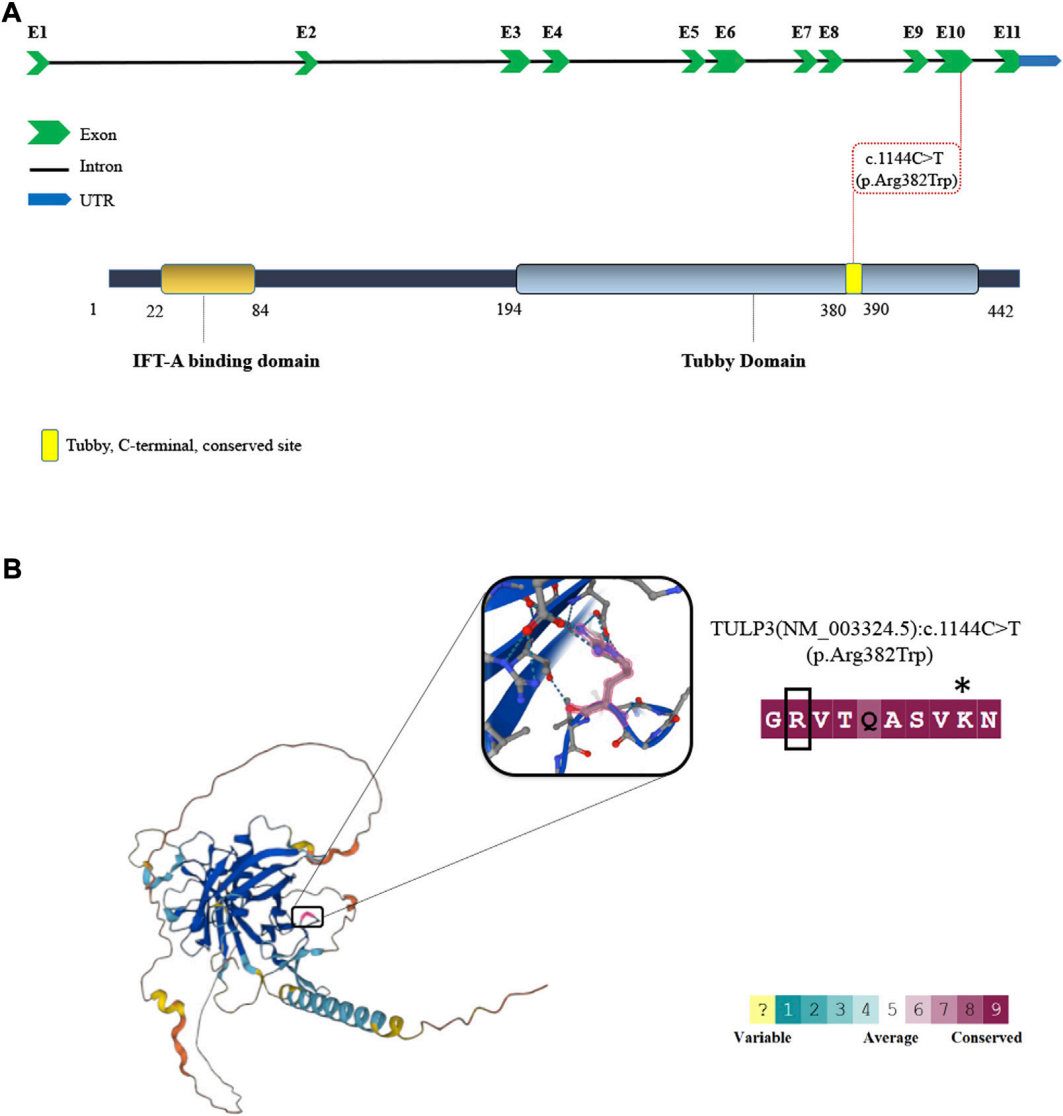
The schematic depiction of *TULP3* mutation. **(A)** Schematic depiction of the human *TULP3* locus with 11 exons (top) and Tubby-like protein 3 (bottom). The red dash line represents the variant in the present study. **(B)** Three-dimensional structure of TULP3 protein and illustration of the conservation scores. The position of the pathogenic variant in this study (pink) are shown on the Protein Structure and illustration of the conservation scores (consurf). * represents the site of lysine to isoleucine missense mutation in the mouse *Tulp3* associated with cystic kidneys (Legue and Liem, 2019).

Variants in *TULP3* have recently been identified in patients with variable and progressive organ fibrosis (Devane et al., 2022). The *TULP3* locus contains 11 exons and encodes a 442 amino acid protein with a N-terminal IFT-A binding domain and a C-terminal Tubby domain (Figure 2A). The mutation affects a highly conserved residue within the Tubby domain substituting the invariant basic arginine to a polar neutral tryptophan, resulting in a change of charge (Figure 2B). This mutation was predicted damaging as assessed by multiple bioinformatics programs. The R382W mutation lies within the Tubby domain which has been shown to be required for the binding of TULP3 to phosphoinositides as well as for the proper trafficking of a subset of membrane-associated proteins into the primary cilium (Santagata et al., 2001; Mukhopadhyay et al., 2010; Badgandi et al., 2017).

**FIGURE 3:**
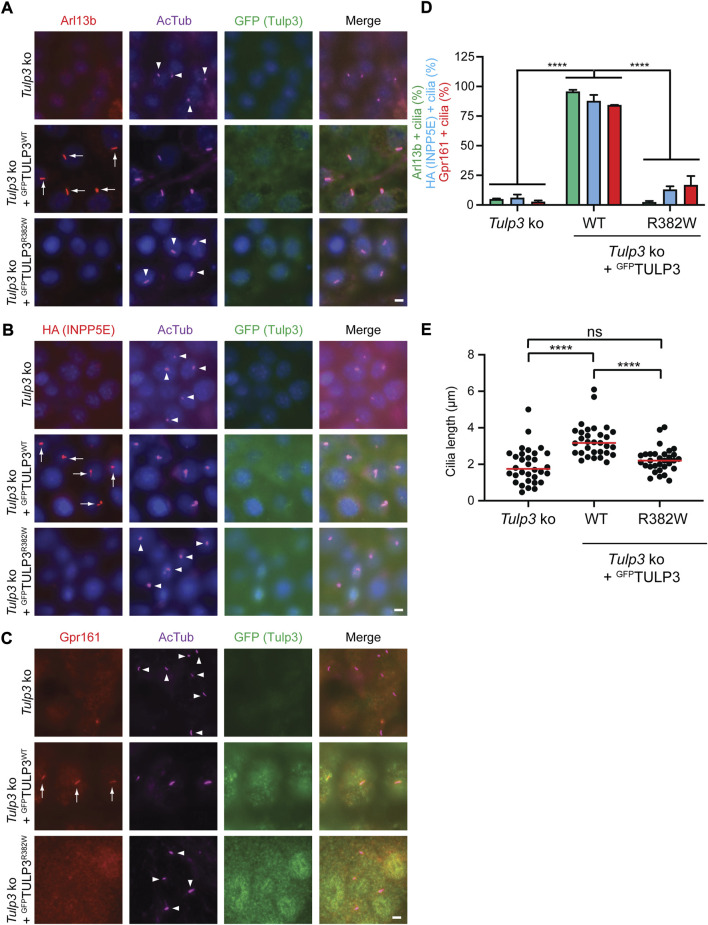
Analysis of the TULP3 R382W variant in ciliary trafficking. **(A)**-C. *Tulp3* ko IMCD3 parental line or lines stably expressing HA-INPP5E and LAP-tagged wild type TULP3 or R382W mutant were grown to confluence, serum starved for 36 h before fixation and immune-stained for Arl13B **(A)**, HA **(B)** or Gpr161 **(C)** and GFP, acetylated tubulin (AcTub) and counterstained for DNA. Arrows and arrowheads indicate cilia positive or negative for the indicated proteins, respectively. Scale = 5 μm. **(D)**. Cilia in GFP(TULP3) expressing cells counted from at least 2 experiments, *n* > 200/condition. Data represents mean ± SD. ****p < 0.0001, two-way ANOVA followed by Tukey multiple comparison test. **(E)**. Cilia lengths (n = 30 cilia/condition). ****p < 0.0001; ns, not significant by using one-way ANOVA followed by Tukey multiple comparison test.

In mice, *Tulp3* mutations disrupt ciliary trafficking leading to abnormal ciliary protein composition, defects in cellular signaling and renal cystic disease (Hwang et al., 2019; Legue and Liem, 2019). To determine how the patient variant R382W affected TULP3 protein function, we analyzed its effect on ciliary trafficking *in vitro*. We generated *Tulp3* knock out (ko) inner medullary collecting duct-3 (IMCD3) cells using CRISPR/Cas9 gene editing methods. We transfected *Tulp3* ko IMCD3 cells with constructs encoding either GFP-tagged TULP3 WT or TULP3 R382W mutant proteins. TULP3 has been shown to facilitate the transport of specific membrane associated proteins into the cilia and IMCD3 cells are routinely used to analyze ciliary trafficking phenotypes. Arl13b, Gpr161, or Inpp5e proteins have all been previously shown to localize to the cilium in a TULP3-dependent manner (Badgandi et al., 2017; Hwang et al., 2019; Legue and Liem, 2019). We first tested the ability of R382W TULP3 to facilitate the ciliary transport of *Arl13b*, a small G-protein associated with Joubert’s Syndrome that requires *Tulp3* for transport into the cilium in kidney cells (Hwang et al., 2019; Legue and Liem, 2019). As expected, Arl13b did not localize to the cilium in *Tulp3* ko IMCD3 cells (Figure 3A). However, transfection of *Tulp3* ko IMCD3 cells with a WT TULP3 construct rescued ciliary transport, as endogenous Arl13b was robustly detected in cilia by immunofluorescence staining (Figure 3A). In contrast, transfection of *Tulp3* ko IMCD3 cells with a R382W TULP3 construct failed to rescue ciliary transport of Arl13b, which was not detected in cilia (Figures 3A,D). This result indicated that the mutant R382W form of TULP3 lacked the ability to facilitate the transport of Arl13b to the cilium.

We next tested the ability of R382W TULP3 to facilitate the transport of Inpp5e into the cilium using *Tulp3* ko IMCD3 cells expressing HA-INPP5E. INPP5E is a ciliary phosphoinositide phosphatase that localizes to cilia where it regulates PI(4)P levels (Kisseleva et al., 2000; Chavez et al., 2015; Garcia-Gonzalo et al., 2015; Jacoby et al., 2009; Bielas et al., 2009) and INPP5E localization into cilia has been shown to be dependent on TULP3 function (Badgandi et al., 2017). *Tulp3* ko IMCD3 cells did not transport HA-tagged INPP5E into the cilium (Figure 3B). However, *Tulp3* ko IMCD3 cells expressing the WT TULP3-GFP rescued transport of HA-tagged INPP5E into cilia as detected by immunofluorescence staining with an antibody to HA (Figure 3B). In contrast, *Tulp3* ko IMCD3 cells expressing R382W mutant TULP3 failed to restore ciliary transport of HA-INPP5E as ciliary levels of HA-INPP5E were significantly reduced compared to WT TULP3 (Figures 3B,D), indicating the mutant form had diminished function.

In addition, we tested the ability of R382W TULP3 to transport the receptor Gpr161 into the cilium. Tulp3 has been previously shown to be required for the transport of numerous G-protein coupled receptors into the cilium, including Gpr161 (Mukhopadhyay et al., 2010; Mukhopadhyay et al., 2013). *Tulp3* ko IMCD3 cells did not transport Gpr161 into the cilium (Figure 3C), however cells expressing WT TULP3 rescued ciliary transport with endogenous Gpr161 localized to cilia as detected by immunofluorescence (Figure 3C). In contrast, *Tulp3* ko IMCD3 cells expressing R382W TULP3 had diminished levels of Gpr161 into the cilium, as cilia contained significantly less Gpr161 (Figures 3C,D). These results again indicated that R382W is a loss of functional allele with a compromised ability to facilitate the transport of membrane-associated proteins into the cilium (Figure 3D).

Finally, we tested the effect of expressing the patient variant R382W form of TULP3 on cilia formation. Cilia in *Tulp3* ko IMCD3 cilia expressing either WT TULP3 or R382W TULP3 were examined by immunofluorescence staining using an anti-acetylated alpha tubulin antibody, which labels the ciliary axonemes. *Tulp3* ko IMCD3 cells expressing the R382W variant form of TULP3 did not affect ciliogenesis but showed a mild reduction in cilia length compared with the same cells expressing WT TULP3, similar to the cilia length reduction in the un-transfected *Tulp3* ko IMCD3 cells (Figure 3E). Taken together, these results indicated that the R382W TULP3 variant identified in patients with fibrocystic renal and hepatic disease caused a loss of function of TULP3 by disrupting protein transport to the primary cilium.

## Discussion

Hepatorenal fibrocystic diseases are inherited disorders that involve developmental and degenerative abnormalities of the liver and kidneys (Gunay-Aygun, 2009). Many of the identified genes that are associated with renal or hepatic cystic and fibrotic diseases encode proteins that are localized to the primary cilium, strongly implicating these signaling organelles in regulating fibrocystic and cystic pathways (Tobin and Beales, 2009; Hildebrandt et al., 2011; Walker et al., 2022). Here we present two sisters with inherited fibrocystic kidney and liver disease attributable to a rare homozygous mutation in the ciliary protein TULP3. The spectrum of phenotypes is consistent with that reported by (Devane et al., 2022), however, our proband had earlier kidney failure than any previously reported case. It is also possible that her deceased siblings had the same genotype and kidney failure even sooner, however insufficient data is available to draw conclusions. Variants in *TULP3* have a variable disease presentation (Devane et al., 2022) and R382W appears to cause relatively severe disease within this spectrum based on the early disease phenotype observed in the proband, although clear phenotypic variability exists among the siblings. Hypertrophic cardiomyopathy in the 6th and 7th decade of life has also been associated with *TULP3* patient variants (Devane et al., 2022). However, the younger patients presented here have an unremarkable clinical cardiac exam and have yet to undergo echocardiography. The recessive inheritance of *TULP3* mutations is also consistent with our genetic studies in the mouse where the conditional knockout of both copies of *Tulp3* in renal epithelial cells drive kidney cysts, while deletion of a single copy of *Tulp3* failed to show phenotypes (Hwang et al., 2019; Legue and Liem, 2019). The results did not support the association of a heterozygous R382W variant in TULP3 with anencephaly (Kuang et al., 2022). Taken together, these findings strongly support the idea the R382W disrupts a key region required for Tubby domain function.

The R382W mutation resides in the Tubby domain of TULP3, a protein motif that binds to phosphoinositides. The Tubby domain has been shown to be required for Tulp3 function to facilitate the trafficking of proteins to the ciliary membrane (Badgandi et al., 2017). The human Tubby domain proteins include TUB, TULP1, TULP2, and TULP3, and are characterized by a conserved C-terminal Tubby domain structurally characterized by a *ß*-barrel surrounding a central alpha helix (Boggon et al., 1999). The Arginine at position 382 is highly conserved in the family of Tubby domain-containing proteins and is present in human as well as *S. cerevisiae* and Arabidopsis (Boggon et al., 1999). The expression of R382W mutant form of TULP3 was unable to rescue ciliary trafficking of Arl13b, INPP5E and Gpr161 in *Tulp3* knockout IMCD3 cells, indicating that the mutation disrupts TULP3 function. This phenotype is reminiscent of earlier studies showing that mutations or deletions of the Tubby domain strongly affected the trafficking of proteins into the cilium (Mukhopadhyay et al., 2010). The R382W amino acid substitution disrupts a similar region of the Tubby domain as the N-ethyl-N-nitrosourea (ENU)-induced missense mutation K407I in the mouse Tulp3 (position 389 in the human protein, Figure 2) (Legue and Liem, 2019). This recessive hypomorphic allele was identified in a forward genetic screen in the mouse that caused rapid cystic renal disease (Legue and Liem, 2019) and the analogous mutation in human TULP3, K389I, disrupted ciliary trafficking of ARL13B, GPR161, and INPP5E (Palicharla et al., 2021).

Patient sequencing studies have also helped to identify critical residues within the Tubby domain that are essential for protein function. Amino acid substitutions within the beta strands have been linked to TULP3-associated disease, including variants C204W and R408H (Devane et al., 2022). Homozygous mutant R408H urine-derived epithelial cells (UREC) also showed ciliary trafficking defects in ARL13B, GPR161 and INPP5E (Devane et al., 2022). In our study, we identified the R382W variant in TULP3 that changes a highly conserved positively charged amino acid with a neutral residue (Figure 2). Interestingly, amino acid substitutions of this conserved arginine within the Tubby domain in the related gene TULP1 have also been identified in retinitis pigmentosa patients (den Hollander et al., 2007; Ajmal et al., 2012). The substitution of this key arginine with a residue that results in a change of charge could alter putative interactions of the Tubby domain or disrupt the 3-dimensional protein structure in this critical region, causing loss of Tubby domain function and disease.

The mechanisms by which mutations in TULP3 drive hepatorenal fibrocystic disease are not understood. However, there is clear evidence that TULP3 facilitates the transport of a critical subset of membrane associated proteins into the primary cilium and that the proper localization of these proteins are thought to be essential for their function (Badgandi et al., 2017). Multiple integral membrane and lipidated proteins are preferentially localized to the cilium (Hilgendorf et al., 2016). These proteins include Arl13b, a Joubert syndrome associated protein which causes cystic renal disease when conditionally deleted in renal epithelial cells in the mouse (Seixas et al., 2016; Li et al., 2016). Deletion of *Inpp5e* in renal epithelial cells also results in strong cystic phenotypes while changing the composition of the ciliary membrane (Bielas et al., 2009). Moreover, Tulp3 has also been shown to regulate the trafficking of other critical disease-associated proteins to cilia such as Polycystins 1 and 2 (mutated in ADPKD) and fibrocystin (mutated in ARPKD) (Badgandi et al., 2017). Therefore, a mutation in the Tubby domain of TULP3 may result in fibrocystic disease in patients by disrupting the transport of multiple signaling proteins regulated by the cilium. A missense mutation in the Tubby domain also results in Sonic Hedgehog dependent phenotypes, a cilia dependent pathway that could also contribute to the disease (Legue and Liem, 2020). However other mechanisms not involving ciliary trafficking, such as TULP3 functions in the nucleus have also been proposed and could regulate fibrocystic pathways (Devane et al., 2022).

In sum, we identified a missense mutation in the gene *TULP3* by WES in two Iranian sisters from a consanguineous family presenting with fibrocystic renal and hepatic disease. The identification of the *TULP3* variant (NM_003324.5: c.1144C>T, p. Arg382Trp) broadens our knowledge of pathogenic mutations in patients and identifies a critical residue within the C-terminal Tubby domain of TULP3 required for proper trafficking of membrane associated proteins to cilia.

## Data Availability

The datasets presented in this study can be found in online repositories. The names of the repository/repositories and accession number(s) can be found in the article/Supplementary Material.
