# Supplementary material for: A pathogenic variant of TULP3 causes renal and hepatic fibrocystic disease
Source: Front Genet. 2022 Oct 7;13:1021037. doi: 10.3389/fgene.2022.1021037 (PMC9585244; doi:10.3389/fgene.2022.1021037)
Supplement: Supplementary file 1 [file Table2.DOCX]

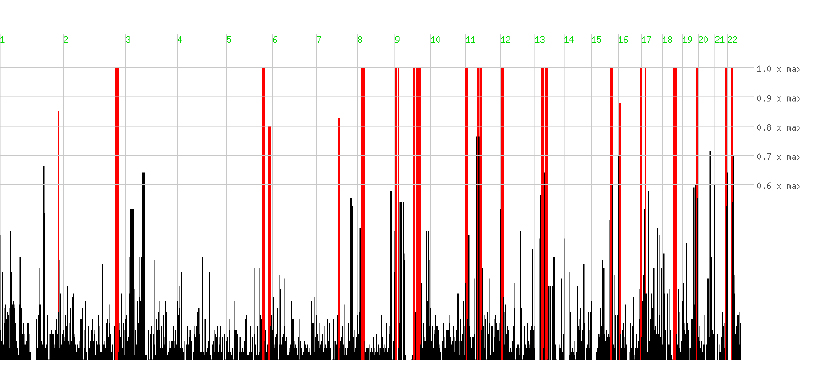


**Supplementary File 1. Homozygosity plot of proband.**

Homozygosity mapping results for the proband (individual VI-4). Homozygosity-mapper plot shows homozygous regions in the proband (red lines).
